# Data-driven analysis of the effect of screening and treatment on the spread of HIV in developing and developed countries

**DOI:** 10.3389/fpubh.2024.1437678

**Published:** 2024-11-14

**Authors:** Wasim Abbas, M. A. Masud, Sajida Parveen, Hyojung Lee, Sangil Kim

**Affiliations:** ^1^Department of Mathematics and Institute of Mathematical Sciences, Pusan National University, Busan, Republic of Korea; ^2^Integrated Mathematical Oncology Department, H Lee Moffitt Cancer Center and Research Institute, Tampa, FL, United States; ^3^Department of Statistics, Kyungpook National University, Daegu, Republic of Korea; ^4^Institute for Future Earth, Pusan National University, Busan, Republic of Korea

**Keywords:** HIV/AIDS, horizontal and vertical transmission, maximum likelihood method, screening, treatment

## Abstract

**Introduction:**

In this study, we used a mathematical epidemic model to explore the status of the HIV epidemic in the USA and Pakistan. In addition to studying the dynamics of the model, we fitted the model with recent data to estimate the parameters describing the epidemic in both countries.

**Results:**

Our estimation shows that in the USA, the reproduction number is 0.9688 (0.9684, 0.9694); if the reproduction number is maintained at this level, it would take a long time to eradicate HIV entirely. Meanwhile, it is 2.2599 (2.2556, 2.2656) in Pakistan, which is due to a lack of awareness in the confirmed group and a lower rate of maintained treatment. We also estimated the rate of vertical transmission, which plays a significant role in Pakistan but not in the USA.

**Discussion:**

We conclude that improving the screening rate and educating people would be effective for controlling HIV in Pakistan, whereas improved screening rate in the USA can eradicate HIV faster.

## 1 Introduction

Human immunodeficiency virus (HIV) attacks the body's immune system, leading to acquired immunodeficiency syndrome (AIDS) in the chronic stage. AIDS is among the most devastating diseases in human history. The dynamics of HIV transmission are influenced by both horizontal (e.g., sexual contact) and vertical (mother-to-child) pathways, and the impact of these pathways varies widely by region. However, awareness, proper treatment, and care can help to control the spread of the disease. Cutting-edge treatment of an infected person may increase lifespan, improve health, and reduce the risk of both types of transmission (1).

The incidence of HIV/AIDS is very high in most developing nations, with some countries experiencing worsening condition daily (2). For instance, HIV cases in Pakistan have increased rapidly in recent years (3). The first HIV-infected person in Pakistan was detected in 1987 (4, 5). The prevalence rate of HIV is still not high in Pakistan, but in recent years, the HIV-infected population has greatly increased in Pakistan. According to UNAIDS (6), from 2010 to 2018, the number of new HIV infections increased from 14,000 to 22,000, while AIDS-related deaths surged by over 350% since 2010. In contrast, developed countries like the USA have seen a decline in new infections due to consistent efforts to improve awareness and treatment. As of 2018, ~84% of the HIV population in the USA had been diagnosed, though one in seven individuals do not know the status of their infection (7). In Pakistan, only 14% of the HIV population knew the status of their infection, and a mere 10% were receiving treatment (6). This stark difference in screening and treatment rates between the USA and Pakistan underscores the need for targeted intervention strategies.

Mathematical modeling plays an important role in understanding epidemic diseases such as HIV. The pioneering mathematical model of HIV was proposed by Anderson and May in 1986 (8–10); this model has been further refined by many researchers (11–22). For instance, in Busenberg et al. (13), the role of sex workers was investigated, and dilution by increasing the number of sex workers was shown to be an effective measure for decreasing HIV incidence. However, this was later criticized, and a decrease in the sex industry with the use of condoms was proven more effective (21). A differential infectivity model stressed the importance of identifying super spreaders through contact tracing besides the use of condoms (15). Contact tracing was also emphasized in the study of De Arazoza and Lounes (23). Another model on a homosexual cohort with differential infectivity showed that reducing the number of partners is key to reducing the incidence of HIV (11).

In addition to horizontal transmission, HIV can also be transmitted vertically to newborns from infected parents. According to UNAIDS (24), 160,000 juveniles (aged 0–14 years) became infected globally in the year 2018 alone. Pakistan is also confronted with the devastating impact of the epidemic on its young population. The number of infected juveniles in Pakistan increased continuously from 2010 to 2018 (25). This may divert health and welfare resources. In Naresh et al. (26), the authors modeled horizontal and vertical transmission explicitly. They considered that a fraction of newborns were HIV-infected and grouped into the infectious class. However, newborns are not infectious, as they do not transfer the disease either horizontally or vertically until adulthood. This was further addressed in López et al. (27), where the authors proposed a two-age group model considering horizontal and vertical transmission, and only the adult infected group was responsible for either type of transmission. In a recent study, the role of vertical transmission was modeled by moving a fraction (proportional to the fraction of infected) of the new recruitment into the infectious class (28). Testing of pregnant women has been recommended to reduce vertical transmission (29).

The role of awareness in controlling HIV and AIDS was modeled in Kaur et al. and Kaymakamzade et al. (30, 31) by considering the transmission rate as an asymptotically decreasing function of the number of infected individuals. The roles of screening and treatment have been recognized by many authors (23, 32–36). The combined role of condom usage, screening, and treatment was investigated using optimal control analysis (33). As treatment increases the lifespan of an infected individual, it may also increase the incidence (27). However, treatment implemented with sufficient awareness is effective in reducing the incidence of HIV (33).

As the disease may be transmitted both horizontally and vertically, treatment should impact the transmission in both ways. Therefore, it is important to also understand the impact of treatment on vertical transmission to deal with the growing number of infected juveniles. However, existing models with vertical transmission are tailored with minor limitations regarding non-infectious juveniles becoming infected (26, 28, 29). In the two-age group model proposed by López et al. (27), only the adult class of infected individuals is considered responsible for transmitting the infection. The prevailing focus of some studies, including those conducted by the authors in Olaniyi et al. (37) and Alhassan et al. (38), is on general horizontal and vertical transmission, overlooking the inclusion of juvenile populations or real-world data, and the absence of country-specific analysis. On the other hand, recent studies conducted by Khan et al. (39) and Teklu and Mekonnen (40) highlighted the significance of incorporating awareness strategies and real-world data into epidemic models. In a similar vein, Teklu and Rao (41) emphasize the importance of identifying key transmission pathways to inform interventions that are effective, especially in settings where resources are limited. In the context of HIV/AIDS, researchers have also investigated delay strategies and stochastic models to achieve insights into the impact of delayed treatment on transmission dynamics. These studies underscore the significance of timely interventions in controlling the epidemic (42, 43). Our study expands upon these existing foundations by integrating awareness and treatment interventions into a compartmental model to determine the effects of these strategies on both horizontal and vertical transmission routes.

Our study further develops these understandings by providing quantitative estimations of the contributions made by both horizontal and vertical transmission pathways to the spread of the HIV epidemic in two distinct socioeconomic contexts—the USA and Pakistan. Based on the information available to us, this study is the first to quantify the relative impacts of these pathways and propose country-specific recommendations grounded in real-world data.

We also modeled the role of treatment as an extension of infectious life, which resulted in a “treat or not to treat” dilemma. The available cutting-edge treatments not only increase life span but also reduce the probability of transmission both vertically and horizontally. Furthermore, by incorporating both horizontal and vertical transmission pathways and distinguishing between treated and untreated infected populations, this work provides a detailed and practical framework for policymakers to optimize interventions suited to their national circumstances.

## 2 Mathematical modeling

We incorporated the heterogeneity in transmission from infected people, and people assumed that people maintained/continued their treatment in the model as proposed by López et al. (27). We denote the size of the total population at time *t* by *N*(*t*), which is divided into a juvenile class *J*(*t*) and adult class *A*(*t*). The juvenile class is further divided into two sub-classes: susceptible juvenile population *J*_1_(*t*) and infected juvenile population *J*_2_(*t*). The adult class is divided into three sub-classes: susceptible adult population *A*_1_(*t*), infected adult population not in treatment *A*_2_(*t*), and infected adult population in treatment *A*_3_(*t*).

We consider both horizontal and vertical transmission. Horizontal transmission occurs when an individual in class *A*_1_ acquires infection from individuals in class *A*_2_ or *A*_3_ by risky acts, such as unprotected sexual intercourse, sharing needles, etc., with a transmission rate *v*_1_ or *v*_2_, respectively, and this individual moves to class *A*_2_. We assume that juveniles are sexually inactive and can only be infected through vertical transmission. Vertical transmission occurs during birth from individuals in *A*_2_ or *A*_3_ with probabilities *r* and ϵ, respectively; the birth rate is β_2_ for both classes. Therefore, the recruitment rate into class *J*_2_ is β_2_(*rA*_2_ + ϵ*A*_3_). The remaining births from *A*_2_ and *A*_3_, β_2_((1 − *r*)*A*_2_ + (1 − ϵ)*A*_3_), are recruited to class *J*_1_. Moreover, susceptible adults give birth to β_1_*A*_1_ susceptible juveniles. Juveniles mature and move to *A*_1_ and *A*_3_ from *J*_1_ and *J*_2_, respectively, at a maturation rate η. Individuals from class *A*_2_ learn their status of infection and start maintaining treatment at a rate θ and accordingly move to class *A*_3_. It is important to note that while specific data related to testing pregnant women are utilized for certain parameters (e.g., ((*r*) and (ϵ)), (θ)) encompasses treatment scenarios for all infected individuals, considering various diagnosis routes. The natural death rates of the juvenile classes (*J*_1_, *J*_2_) and adult classes (*A*_1_, *A*_2_, *A*_3_) are μ_1_ and μ_2_, respectively. We assume that the infected individuals in *A*_3_ maintain treatment, and as a result, the disease-related death rate is negligible. However, the disease-related death rate for individuals in *J*_2_ and *A*_2_ is α.

**Figure 1:**
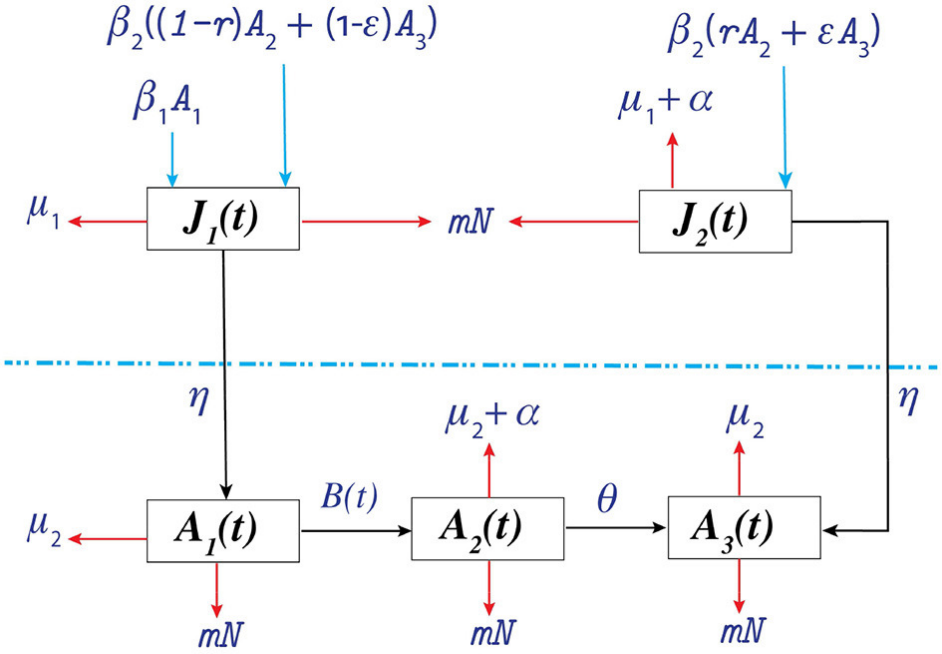
Schematic diagram of the proposed model. $B\left(t\right)=\frac{v_{1}A_{1}A_{2}+v_{2}A_{1}A_{3}}{A}$. The juvenile and adult populations are separated by the dotted line.

We propose juveniles play no significant role in HIV transmission, a claim that is supported by the extensive South African Petra Study (44). The study effectively illustrated that individuals under the age of 15 handled a mere 2% of all HIV transmissions, underscoring the limited autonomous contribution of juveniles in fueling the HIV epidemic. Our modeling approach aligns closely with the guidelines established by the World Health Organization on Mother-to-Child Transmission (MTCT) (45), which emphasize interventions to prevent HIV transmission from infected mothers to their children. This perfectly aligns with our modeling assumption, emphasizing the significance of maternal transmission. Infected juveniles are assumed to transition into infected adults who receive treatment upon reaching adulthood. This assumption aligns with our specific scenario and contributes to our study. The density-dependent death rate is *mN* for all classes. A schematic diagram of the model is shown in Figure 1.

Following the aforementioned assumptions, we may express the model as follows:


(1) $$\begin{array}{l} \frac{dJ_{1}}{dt}=\beta_{1}A_{1}+\beta_{2}\left(\left(1-r\right)A_{2}+\left(1-\epsilon \right)A_{3}\right)-\left(\eta +\mu_{1}+mN\right)J_{1} \end{array}$$

(2) $$\begin{array}{l} \frac{dA_{1}}{dt}=\eta J_{1}-\frac{\left(v_{1}A_{1}A_{2}+v_{2}A_{1}A_{3}\right)}{A}-\left(\mu_{2}+mN\right)A_{1} \end{array}$$

(3) $$\begin{array}{l} \frac{dJ_{2}}{dt}=\beta_{2}\left(rA_{2}+\epsilon A_{3}\right)-\left(\alpha +\eta +\mu_{1}+mN\right)J_{2} \end{array}$$

(4) $$\begin{array}{l} \frac{dA_{2}}{dt}=\frac{\left(v_{1}A_{1}A_{2}+v_{2}A_{1}A_{3}\right)}{A}-\left(\theta +\mu_{2}+\alpha +mN\right)A_{2} \end{array}$$

(5) $$\begin{array}{l} \frac{dA_{3}}{dt}=\eta J_{2}+\theta A_{2}-\left(\mu_{2}+mN\right)A_{3} \end{array}$$

where *A* = *A*(*t*) = *A*_1_(*t*)+*A*_2_(*t*)+*A*_3_(*t*) and *N* = *N*(*t*) = *J*_1_(*t*)+*J*_2_(*t*)+*A*_1_(*t*)+*A*_2_(*t*)+*A*_3_(*t*). We observe that *B*(*t*) is continuous at (*A*_1_, *A*_2_, *A*_3_) = (0, 0, 0) if we define *B*(0, 0, 0) = 0. We also notice that *B* ≤ 2max(*A*_1_, *A*_2_, *A*_3_), and is Lipschitzian for *A*_1_ ≥ 0, *A*_2_ ≥ 0 and *A*_3_ ≥ 0. However, *B*(*t*) is clearly not differentiable at (0, 0, 0).

In our mathematical model, we incorporate a density-dependent death rate (*mN*) for all classes, a simplification commonly utilized in epidemiological models. This assumption serves to represent the impact of population density on mortality rates and imposes bounded growth. It is important to note that such modeling choices involve simplifications for mathematical tractability.

## 3 Model analysis

We performed a mathematical analysis of the model Equations 1–5 to understand its dynamics from an epidemiological perspective.

### 3.1 Equilibria

Our model exhibits one trivial equilibrium (0, 0, 0, 0, 0), a disease-free equilibrium $\left(J_{1}^{*},A_{1}^{*},0,\;0,\;0\right)$, a susceptible extinction equilibrium $\left(0,\;0,J_{2}^{*},0,A_{3}^{*}\right)$, and an endemic equilibrium $\left(J_{1}^{**},A_{1}^{**},J_{2}^{**},A_{2}^{**},A_{3}^{**}\right)$. As *B*(*t*) is not differentiable at the trivial equilibrium, stability analysis at this equilibrium is unfeasible using standard linearization techniques. The criteria for the existence of the remaining equilibria are summarized in the following propositions.

**Proposition 1**. *For the system of* Equations 1–5, *if* $\kappa_{1}=\frac{\beta_{1}\eta }{\mu_{2}\left(\eta +\mu_{1}\right)}>1$, *then the system has a unique disease-free equilibrium* $\left(J_{1}^{*},A_{1}^{*},0,\;0,\;0\right)$*, where*


$$\begin{array}{l} J_{1}^{*}\;=\;\frac{a_{2}N_{1}^{*}}{\eta \;+\;a_{2}},\;A_{1}^{*}\;=\;\frac{\eta N_{1}^{*}}{\eta \;+\;a_{2}} \end{array}$$

*and*


$$\begin{array}{l} N_{1}^{*}=\\ \frac{-\left(\eta +\mu_{1}+\mu_{2}\right)+\sqrt{{\left(\eta +\mu_{1}+\mu_{2}\right)}^{2}-4\mu_{2}\left(\eta +\mu_{1}\right)\left(1-\kappa_{1}\right)}}{2m} \end{array}$$

**Proposition 2**. *For the system of* Equations 1–5, *if* *r* = 1, ϵ = 1 *and*$\kappa_{2}=\frac{\beta_{2}\eta }{\mu_{2}\left(\alpha +\eta +\mu_{1}\right)}>1$, *then this system has a unique susceptible extinction equilibrium* $\left(0,\;0,J_{2}^{*},0,A_{3}^{*}\right)$. *Here*,


$$\begin{array}{l} J_{1}^{*}\;=\;\frac{a_{2}N_{1}^{*}}{\eta \;+\;a_{2}},\;A_{1}^{*}\;=\;\frac{\eta N_{1}^{*}}{\eta \;+\;a_{2}}\\ J_{2}^{**}=\frac{a_{4}a_{5}\left(T_{1}+T_{2}T_{5}\right)\left(\Omega_{1}-\left(1-T_{2}\right)\right)A^{**}}{\Omega_{1}\Omega_{2}\eta },\\ A_{2}^{**}=\frac{a_{5}\left(1-T_{2}\right)\left(\Omega_{1}-\left(1-T_{2}\right)\right)A^{**}}{\Omega_{1}\Omega_{2}}, \end{array}$$

*and*


$$\begin{array}{l} N_{2}^{*}=\frac{-\left(\eta +\mu_{1}+\mu_{2}+\alpha \right)+\sqrt{\kappa_{3}}}{2m} \end{array}$$

*with*


$$\begin{array}{l} \kappa_{3}={\left(\eta +\mu_{1}+\mu_{2}+\alpha \right)}^{2}-4\mu_{2}\left(\alpha +\eta +\mu_{1}\right)\left(1-\kappa_{2}\right). \end{array}$$

**Proposition 3**. *For the system of* Equations 1–5, *if* *T*_2_ < 1 *and* *R*_0_ > 1, *then the system has a unique endemic equilibrium* $\left(J_{1}^{**},A_{1}^{**},J_{2}^{**},A_{2}^{**},A_{3}^{**}\right)$*, where*


$$\begin{array}{l} J_{1}^{**}=\frac{a_{5}\left(1-T_{2}\right)\left(\Omega_{2}+a_{4}\left(\Omega_{1}-\left(1-T_{2}\right)\right)\right)A^{**}}{\Omega_{1}\Omega_{2}\eta },\\ A_{1}^{**}=\frac{\left(1-T_{2}\right)A^{**}}{\Omega_{1}}, \end{array}$$

*and*


$$\begin{array}{l} A_{3}^{**}=\frac{a_{4}\left(T_{1}+T_{5}\right)\left(\Omega_{1}-\left(1-T_{2}\right)\right)A^{**}}{\Omega_{1}\Omega_{2}} \end{array}$$

*with*


$$\begin{array}{l} A=A_{1}+A_{2}+A_{3},\;\Omega_{1}=T_{3}\left(1-T_{2}\right) \end{array}$$

+*T*_4_(*T*_1_ + *T*_5_)*, and* Ω_2_ = *a*_5_(1 − *T*_2_) + *a*_4_(*T*_1_ + *T*_5_).

*T*_1_, *T*_2_, *T*_3_, *T*_4_, *T*_5_, *a*_1_, *a*_2_, *a*_3_, *a*_4_, and *a*_5_ are defined in **Section 3.2**.

Parameter (κ_1_) is a critical factor in Proposition 1, influencing the existence of a unique disease-free equilibrium. Parameter κ_1_: offspring number of susceptible adults.

Parameter (κ_2_) plays a crucial role in Proposition 2, determining the existence of a unique susceptible extinction equilibrium. Parameter κ_2_: basic offspring number of infected adults.

### 3.2 Basic reproduction number R_0_

The basic reproduction number (*R*_0_) is the expected number of infections produced by an infected individual in an entirely susceptible population throughout their entire infectious lifetime.

Following the approach introduced by Van den Driessche and Watmough (46), the next-generation matrix is given by


$$\begin{array}{l} K=\left(\begin{array}{ccc} \frac{\epsilon \beta_{2}\eta }{a_{3}a_{5}} & \frac{\beta_{2}\left(ra_{5}+\epsilon \theta \right)}{a_{4}a_{5}} & \frac{\epsilon \beta_{2}}{a_{5}}\\ \frac{\eta v_{2}}{a_{3}a_{5}} & \frac{v_{1}}{a_{4}}+\frac{v_{2}\theta }{a_{4}a_{5}} & \frac{v_{2}}{a_{5}}\\ 0 & 0 & 0 \end{array}\right) \end{array}$$

where η+μ_1_ + *mN* = *a*_1_, μ_2_+*mN* = *a*_2_, α+η+μ_1_+*mN*=*a*_3_, θ+μ_2_+α+*mN* = *a*_4_, and, μ_2_+*mN* = *a*_5_ = *a*_2_.

The basic reproduction number is the spectral radius $B_{1}=\left(\begin{array}{cc} -\left(a_{1}+mJ_{1}^{*}\right) & \beta_{1}-mJ_{1}^{*}\\ \eta -mA_{1}^{*} & -\left(a_{2}+mA_{1}^{*}\right) \end{array}\right)$ of *K*. Thus,


$$\begin{array}{l} R_{0}=\frac{1}{2}\\ \left(\left(T_{2}+T_{3}+T_{4}T_{5}\right)+\sqrt{{\left(T_{2}-T_{3}-T_{4}T_{5}\right)}^{2}+4T_{4}\left(T_{2}T_{5}+T_{1}\right)}\right) \end{array}$$

where $T_{1}=\frac{r\beta_{2}\eta }{a_{3}a_{4}}$, $T_{2}=\frac{\epsilon \beta_{2}\eta }{a_{3}a_{5}}$, $T_{3}=\frac{v_{1}}{a_{4}}$, $T_{4}=\frac{v_{2}}{a_{5}}$, and $T_{5}=\frac{\theta }{a_{4}}$. Although the expression for *R*_0_ is too complex to interpret, *T*_1_, *T*_2_, …, *T*_5_ produce a meaningful interpretation. The average infectious lifetimes of an infected individual in *A*_2_ and *A*_3_ are 1/*a*_4_ and 1/*a*_5_, respectively. The average lifetime of an infected juvenile individual is 1/*a*_3_; thus, the maturation probability of an infected juvenile is η/*a*_3_. Hence, one infected individual from *A*_2_ transmits the disease vertically to $r\beta_{2}\times \frac{\eta }{a_{3}}\times \frac{1}{a_{4}}=T_{1}$ individuals on average throughout their infectious lifetime. Similarly, one infected individual from *A*_3_ transmits the infection to $\epsilon \beta_{2}\times \frac{\eta }{a_{3}}\times \frac{1}{a_{5}}=T_{2}$ individuals vertically. An infected individual belonging to *A*_2_ transmits the disease to $\frac{v_{1}}{a_{4}}=T_{3}$ individuals on average horizontally. In addition, an infected individual belonging to *A*_3_ transmits the disease to $\frac{v_{2}}{a_{5}}\times \frac{\theta }{a_{4}}=T_{4}\times T_{5}$ individuals horizontally over their infectious lifetime. Thus, *R*_0_ consists of the reproduction numbers associated with the two transmission pathways from each of the two infectious classes *A*_2_ and *A*_3_.

Another threshold of interest is $R_{1}=\frac{\beta_{1}\eta }{a_{1}a_{2}}$, which is the average number of new *J*_1_(*t*) produced by each *A*_1_(*t*) during adulthood multiplied by the probability of the survival of each *J*_1_(*t*) during juvenility.

### 3.3 Stability analysis

In this subsection, we discuss the stability analysis of our system of Equations 1–5. We describe the local and global stability results for the aforementioned non-trivial equilibria. All threshold values are defined in Table 1, and the conditions are biologically meaningful. We present a series of key theorems that establish the stability properties of the system of Equations 1–5 under various conditions.

**Table 1:** All results proved for the system of Equations 1–5.

	**Condition**	**Result**
i.	κ_1_ > 1 and *R*_0_ < 1	Disease-free equilibrium is LAS
ii.	κ_1_ ≤ 1 ⇒ *R*_1_ < 1 and *R*_0_ ≥ 1 with *r* = ϵ = 1	Susceptible extinction equilibrium is LAS
iii.	$\kappa_{1}>\frac{\left(\eta +\mu_{1}\right)\left(v_{1}+v_{2}+\mu_{2}\right)}{\eta \mu_{2}}$ and *T*_1_ + *T*_2_ + *T*_3_ + *T*_4_ + *T*_5_ < 1	Disease-free equilibrium is GS
iv.	*r* = ϵ = 1, κ_1_ ≤ 1 ⇒ *R*_1_ < 1 and $\kappa_{2}>\frac{\eta +\mu_{1}+\alpha }{\eta }$	Susceptible extinction equilibrium is GS
v.	*r* = ϵ = 1, κ_1_ ≤ 1⇒*R*_1_ < 1 and *R*_0_ < 1	Trivial equilibrium is GS

Here, LAS means locally asymptotically stable, and GS means globally stable.

#### 3.3.1 Local stability analysis

**Theorem 1**. *If* κ_1_ > 1 *and* *R*_0_ < 1*, we demonstrate that the disease-free equilibrium is locally asymptotically stable within* ${\mathbb{R}}_{+}^{5}$.

Proof. If κ_1_ > 1, then Proposition 1 ensures the uniqueness of the disease-free equilibrium. Now, for stability analysis, the Jacobian of the mathematical model represented by Equations 1–5 and evaluated at the disease-free equilibrium is:


$$\begin{array}{l} B=\left(\begin{array}{cc} B_{1} & B_{3}\\ O & B_{2} \end{array}\right) \end{array}$$

where


$$\begin{array}{l} B_{2}=\left(\begin{array}{ccc} -a_{3} & r\beta_{2} & \epsilon \beta_{2}\\ 0 & v_{1}-a_{4} & v_{2}\\ \eta & \theta & -a_{5} \end{array}\right) \end{array}$$

$$\begin{array}{l} B_{3}=\left(\begin{array}{ccc} -mJ_{1}^{*} & \left(1-r\right)\beta_{2}-mJ_{1}^{*} & \left(1-\epsilon \right)\beta_{2}-mJ_{1}^{*}\\ -mA_{1}^{*} & -\left(v_{1}+mA_{1}^{*}\right) & -\left(v_{2}+mA_{1}^{*}\right) \end{array}\right) \end{array}$$

and $O=\left(\begin{array}{cc} 0 & 0\\ 0 & 0\\ 0 & 0 \end{array}\right)$.

The characteristic polynomial of *B*_1_, denoted by *charpoly*(*B*_1_), is given as


$$\begin{array}{l} \text{charpoly}\left(B_{1}\right)=\lambda^{2}+b_{1}\lambda +b_{2}\text{,} \end{array}$$

where $b_{1}=a_{1}+a_{2}+mA_{1}^{*}+mJ_{1}^{*}$ and $b_{2}=a_{1}a_{2}-\beta_{1}\eta +a_{1}mA_{1}^{*}+\beta_{1}mA_{1}^{*}+a_{2}mJ_{1}^{*}+\;\eta mJ_{1}^{*}.$

From Proposition 1 and Equations 1–5, we note that ηβ_1_ = *a*_1_*a*_2_. This implies that all the coefficients of *charpoly*(*B*_1_) are positive. Hence, by the Routh–Hurwitz stability criteria, all eigenvalues of *B*_1_ have negative real parts.

Moreover, $charpoly\left(B_{2}\right)=\lambda^{3}+c_{1}\lambda^{2}+c_{2}\lambda +c_{3},$ where *c*_1_ = *a*_3_ + *a*_5_ + *a*_4_(1 − *T*_3_), *c*_2_ = *a*_3_*a*_4_(1 − *T*_3_) + *a*_3_*a*_5_(1 − *T*_2_) + *a*_4_*a*_5_(1 − *T*_3_ − *T*_4_*T*_5_), and *c*_3_ = *a*_3_*a*_4_*a*_5_(*T*_2_*T*_3_ + 1 − *T*_2_ − *T*_3_ − *T*_1_*T*_4_ − *T*_4_*T*_5_).

Because *R*_0_ < 1 by assumption, we have *c*_1_ > 0, *c*_3_ > 0, and *c*_1_*c*_2_ > *c*_3_. Thus, according to the Routh–Hurwitz stability criteria, all the eigenvalues have negative real parts. This implies that the disease-free equilibrium is locally asymptotically stable.

The local asymptotic stability of the disease-free equilibrium suggests that the disease will eventually be eliminated from the population sufficiently close to disease-free equilibrium when *R*_0_ < 1 and κ_1_ > 1. Within this context, the transmission of HIV is at a minimal level, and the implemented control measures, encompassing treatment, preventive interventions, and public health strategies, demonstrate sufficient efficacy in averting the prolonged presence of the virus. The system reverts to a state devoid of disease, signifying the successful eradication of HIV within the population.

**Theorem 2**. *Under specific conditions involving* *R*_1_ < 1 *and* κ_2_ > 1(⇒*R*_0_ ≥ 1) *with* *r* = ϵ = 1*, we establish the local asymptotic stability of the susceptible extinction equilibrium in the domain* ${\mathbb{R}}_{+}^{5}.$

Proof. If κ_2_ > 1, then Proposition 2 ensures the uniqueness of the susceptible extinction equilibrium. For the stability analysis of this equilibrium, the Jacobian of the mathematical model (Equations 1–5) evaluated at the susceptible extinction equilibrium is:


$$\begin{array}{l} D=\left(\begin{array}{cc} D_{1} & D_{3}\\ O & D_{2} \end{array}\right). \end{array}$$

Here,


$$\begin{array}{l} D_{1}=\left(\begin{array}{cc} -a_{1} & \beta_{1}\\ \eta & -\left(v_{2}+a_{2}\right) \end{array}\right) \end{array}$$

$D_{2}=\left(\begin{array}{ccc} -\left(a_{3}+mJ_{2}^{*}\right) & \beta_{2}-mJ_{2}^{*} & \beta_{2}-mJ_{2}^{*}\\ 0 & -a_{4} & 0\\ \eta -mA_{3}^{*} & \theta -mA_{3}^{*} & -\left(a_{5}+mA_{3}^{*}\right) \end{array}\right)$,


$$\begin{array}{l} D_{3}=\left(\begin{array}{cc} -mJ_{2}^{*} & -mJ_{2}^{*}\\ 0 & v_{2}\\ -mA_{3}^{*} & -mA_{3}^{*} \end{array}\right) \end{array}$$

and $O=\left(\begin{array}{ccc} 0 & 0 & 0\\ 0 & 0 & 0 \end{array}\right)$.

The characteristic polynomial of *D*_1_ denoted by *charpoly*(*D*_1_) is given as


$$\begin{array}{l} \text{charpoly}\left(D_{1}\right)=\lambda^{2}+d_{1}\lambda +d_{2}\text{,} \end{array}$$

while *d*_1_ = *a*_1_ + *a*_2_ + *v*_2_ and *d*_2_ = *a*_1_*a*_2_ − β_1_η + *a*_1_*v*_2_.

From supposition *R*_1_ < 1, this implies that *a*_1_*a*_2_ − ηβ_1_ > 0. Thus, all the coefficients of *charpoly*(*D*_1_) are positive.

Moreover, *charpoly*(*D*_2_) = *f*_1_(λ)·*f*_2_(λ).

Here,


$$\begin{array}{l} f_{1}\left(\lambda \right)=\lambda +a_{4} \end{array}$$

and


$$\begin{array}{l} f_{2}\left(\lambda \right)=\lambda^{2}+\left(a_{3}+a_{5}+mA_{3}^{*}+mJ_{2}^{*}\right)\lambda +\left(a_{3}+\beta_{2}\right)mA_{3}^{*}\\ +\left(a_{5}+\eta \right)mJ_{2}^{*}+a_{3}a_{5}-\eta \beta_{2} \end{array}$$

From Proposition 2, we notice that ηβ_2_ = *a*_3_*a*_5_; therefore all the coefficients of *f*_2_(λ) are positive. Thus, according to the Routh–Hurwitz stability criteria, all the eigenvalues have negative real parts. This implies that the susceptible extinction equilibrium is locally asymptotically stable.

The local stability analysis of the susceptible extinction equilibrium indicates that, given certain conditions (*R*_1_ < 1, κ_2_ > 1), the susceptible individuals will be eradicated from the population, leaving only the infected individuals. This scenario illustrates the worst possible outcome, in which HIV becomes endemic in the population due to inadequate control measures, resulting in unrestricted virus transmission.

#### 3.3.2 Global stability analysis

**Theorem 3**. *For cases where* $\kappa_{1}>\frac{\left(\eta +\mu_{1}\right)\left(v_{1}+v_{2}+\mu_{2}\right)}{\eta \mu_{2}}$ *and* *R*_0_ < 1*, then* N(*t*) > 0 *(Trivial equilibrium is a Repeller) and the disease-free equilibrium is a global attractor in* ${\mathbb{R}}_{+}^{5}$.

Proof. To prove this theorem, we claim that N(*t*) > 0; on the contrary, suppose that N(*t*) = 0. Let us consider a function *x* = *A*_1_ + ξ_1_*J*_1_, where 0 < ξ_1_ < 1. Differentiating *x* with respect to *t*, along with the solution of the system of Equations 1–5, we obtain,


$$\begin{array}{l} x^{\prime }=\left(\eta J_{1}-a_{2}A_{1}\right)+\xi_{1}\left(\beta_{1}A_{1}-a_{1}J_{1}\right)\\ \geq \left(\frac{\eta }{\xi_{1}}-\left(\eta +\mu_{1}\right)\right)\xi_{1}J_{1}+\left(\frac{\xi_{1}\beta_{1}\eta }{\eta +\mu_{1}}-v_{1}-v_{2}-\mu_{2}\right)A_{1}-mxN\\ =\left(\frac{\eta }{\xi_{1}}-\left(\eta +\mu_{1}\right)\right)\xi_{1}J_{1}+\left(\xi_{1}\mu_{2}\kappa_{1}-v_{1}-v_{2}-\mu_{2}\right)A_{1}-mxN. \end{array}$$

Because $\kappa_{1}>\frac{\left(\eta +\mu_{1}\right)\left(v_{1}+v_{2}+\mu_{2}\right)}{\eta \mu_{2}}$, ∃ ϵ ∈ (0, 1) such that


$$\begin{array}{l} \eta \mu_{2}\kappa_{1}>\left(\eta +\mu_{1}+\epsilon \right)\left(v_{1}+v_{2}+\mu_{2}+\epsilon \right). \end{array}$$

Let us choose ξ_1_ such that,


$$\begin{array}{l} \frac{\left(v_{1}+v_{2}+\mu_{2}+\epsilon \right)}{\mu_{2}\kappa_{1}}<\xi_{1}<\frac{\eta }{\eta +\mu_{1}+\epsilon }. \end{array}$$

Thus, $x^{\prime }>\epsilon \xi_{1}J_{1}+\epsilon A_{1}-mxN=\epsilon x-mxN$.

Now, if N(*t*) = 0, then we observe that *x* → ∞. This contradicts the fact that *x* is bounded. Thus, N(*t*) > 0.

Next, we consider the positive definite function,


$$\begin{array}{l} V\left(X\right)=\sigma_{1}J_{2}+\sigma_{2}A_{2}+\sigma_{3}A_{3},\\ X=\left(J_{1},A_{1},J_{2},A_{2},A_{3}\right)\in {\mathbb{R}}_{+}^{5}\sigma_{1}=\frac{\eta v_{2}}{a_{3}a_{5}}=\frac{\eta T_{4}}{a_{3}} \end{array}$$

with, $\sigma_{2}=1-\frac{\epsilon \beta_{2}\eta }{a_{3}a_{5}}=1-T_{2}$, and $\sigma_{3}=\frac{v_{2}}{a_{5}}=T_{4}$.

Because *R*_0_ < 1, we derive that *T*_2_ < 1 ⇒ 1 − *T*_2_ > 0. Thus,


$$\begin{array}{l} \dot{V}=\frac{\eta T_{4}}{a_{3}}\left(r\beta_{2}A_{2}+\epsilon \beta_{2}A_{3}-a_{3}J_{2}\right)\\ +\left(1-T_{2}\right)\left(\frac{v_{1}A_{1}A_{2}+v_{2}A_{1}A_{3}}{A}-a_{4}A_{2}\right)\\ +T_{4}\left(\eta J_{2}+\theta A_{2}-a_{5}A_{3}\right)\\ \leq \left(\frac{r\beta_{2}\eta T_{4}}{a_{3}}+v_{1}\left(1-T_{2}\right)+\theta T_{4}-a_{4}\left(1-T_{2}\right)\right)A_{2}\\ +\left(\frac{\epsilon \beta_{2}\eta T_{4}}{a_{3}}+v_{2}\left(1-T_{2}\right)-a_{5}T_{4}\right)A_{3}\\ =a_{4}\left(T_{1}T_{4}+T_{3}\left(1-T_{2}\right)+T_{4}T_{5}-\left(1-T_{2}\right)\right)A_{2}\\ +a_{5}\left(T_{2}T_{4}+T_{4}\left(1-T_{2}\right)-T_{4}\right)A_{3}\\ =a_{4}\left(T_{1}T_{4}+T_{3}-T_{2}T_{3}+T_{4}T_{5}-1+T_{2}\right)A_{2}\\ =a_{4}\left(T_{1}T_{4}+T_{2}+T_{3}+T_{4}T_{5}-T_{2}T_{3}-1\right)A_{2}. \end{array}$$

Based on our supposition that *R*_0_ < 1 ⇒ *T*_1_*T*_4_ + *T*_2_ + *T*_3_ + *T*_4_*T*_5_ < 1 + *T*_2_*T*_3_, we have $\dot{V}\leq 0$. This implies that *V* is a Lyapunov function with selected σ_1_, σ_2_, and σ_3_. Thus, according to the Lyapunov–LaSalle invariance principle,


$$\begin{array}{l} E=\left\{X\in {\mathbb{R}}_{+}^{5}|\dot{V}=0\right\}=\left\{\left(J_{1},A_{1,}0,0,0\right)|J_{1}\geq 0,A_{1}\geq 0\right\} \end{array}$$

is an invariant set. Consequently, the largest invariant set is *E* itself. Therefore, all positive solutions of the system of Equations 1–5 tend to the set *E*.

Let us consider the limiting system,


$$\begin{array}{l} J_{1}^{\prime }=\beta_{1}A_{1}-\left(\eta +\mu_{1}\right)J_{1}-\left(J_{1}+A_{1}\right)mJ_{1}=F_{1}\left(J_{1},A_{1}\right) \end{array}$$

(6) $$\begin{array}{l} A_{1}^{\prime }=\eta J_{1}-\mu_{2}A_{1}-mA_{1}\left(J_{1}+A_{1}\right)=F_{2}\left(J_{1},A_{1}\;\right). \end{array}$$

Let $f\left(J_{1},A_{1}\right)=\frac{1}{J_{1}A_{1}}.$ Then, $\frac{\partial \left(fF_{1}\right)}{\partial J_{1}}+\frac{\partial \left(fF_{2}\right)}{\partial A_{1}}<0$ $\forall \;\left(J_{1},A_{1}\right)\in {\mathbb{R}}_{+}^{2}$. By applying the Bendixson–Dulac criteria, we can say that there are no non-trivial periodic orbits in ${\mathbb{R}}_{+}^{2}$. Solutions of this limited system (6) are bounded, and there is only one non-trivial positive equilibrium point. Because trivial equilibrium is repellent in the system of Equations 1–5, it is also repellent in the system (6). In addition, Theorem 1 shows the local stability of the positive steady state of the system (6). Thus, from these two results, we conclude that the ω-limit set of bounded positive solutions of the system (6) is only $\left(J_{1}^{*},A_{1}^{*}\right)$. Hence, for the system of Equations 1–5, the disease-free equilibrium $\left(J_{1}^{*},A_{1}^{*},0,\;0,\;0\right)$ is a global attractor in ${\mathbb{R}}_{+}^{5}$.

The global stability of the disease-free equilibrium reveals that, irrespective of disparate initial infection levels, the population will ultimately attain a disease-free state, if *R*_0_ < 1 and κ_1_ exceeds a certain threshold. From a biological standpoint, it can be concluded that the implementation of comprehensive HIV control measures, including extensive testing, treatment, and prevention strategies, holds the potential for effectively eradicating HIV within the population. This global stability result underscores the importance of maintaining strong public health interventions.

**Theorem 4** *If* *r* = ϵ = 1, *R*_1_ < 1*, and* $\kappa_{2}>\frac{\eta +\mu_{1}+\alpha }{\eta }$, we establish that *the susceptible extinction equilibrium is a global attractor in* ${\mathbb{R}}_{+}^{5}$.

Proof. First, we claim that N(*t*) > 0 under the aforementioned assumptions. On the contrary, assume that N(*t*) = 0. Let us suppose a function *y* = *A*_3_ + ξ_2_*J*_2_ with 0 < ξ_2_ < 1. The derivative *y* along with the solution of the system, Equations 1–5, with respect to *t* will be,


$$\begin{array}{l} y^{\prime }=\left(\eta J_{2}-a_{5}A_{3}\right)+\xi_{2}\left(\beta_{2}A_{3}-a_{3}J_{2}\right)\\ =\left(\eta J_{2}-\mu_{2}A_{3}\right)+\xi_{2}\left(\beta_{2}A_{3}-\left(\eta +\mu_{1}+\alpha \right)J_{2}\right)-myN\\ \geq \left(\frac{\eta }{\xi_{2}}-\left(\eta +\mu_{1}+\alpha \right)\right)\xi_{2}J_{2}+\left(\frac{\xi_{2}\beta_{2}\eta }{\eta +\mu_{1}+\alpha }-\mu_{2}\right)A_{3}-myN. \end{array}$$

Because $\kappa_{2}>\frac{\eta +\mu_{1}+\alpha }{\eta }=\frac{\mu_{2}\left(\eta +\mu_{1}+\alpha \right)}{\eta \mu_{2}},\;\exists$ $\tilde{\epsilon }\in \left(0,\;1\right)$ such that


$$\begin{array}{l} \eta \mu_{2}\kappa_{2}>\left(\eta +\mu_{1}+\alpha +\tilde{\epsilon }\right)\left(\mu_{2}+\tilde{\epsilon }\right). \end{array}$$

Let ξ_2_ be chosen such that,

$\frac{\left(\mu_{2}+\tilde{\epsilon }\right)}{\mu_{2}\kappa_{2}}<\xi_{2}<\frac{\eta }{\left(\eta +\mu_{1}+\alpha +\tilde{\epsilon }\right)}<\;1$.

Then, $y^{\prime }>\tilde{\epsilon }\xi_{2}J_{2}+\tilde{\epsilon }A_{3}-myN=\tilde{\epsilon }y-myN$. If N(*t*) = 0, then *y* → ∞, which contradicts the fact that *y* = *A*_3_ + ξ_2_*J*_2_ < *N*. Therefore, N(*t*) > 0.

Now, we consider the positive definite function $V\left(X\right)=\varrho J_{1}+A_{1}+A_{2},X=\left(J_{1},A_{1},J_{2},A_{2},A_{3}\right)\;{\in \mathbb{R}}_{+}^{5}$.

Setting $\varrho =\frac{\eta }{a_{1}}$, we have


$$\begin{array}{l} \dot{V}\left(X\right)=\frac{\eta }{a_{1}}\left(\beta_{1}A_{1}-a_{1}J_{1}\right)+\left(\eta J_{1}-\frac{v_{1}A_{1}A_{2}+v_{2}A_{1}A_{3}}{A}-a_{2}A_{1}\right)\\ +\left(\frac{v_{1}A_{1}A_{2}+v_{2}A_{1}A_{3}}{A}-a_{4}A_{2}\right)\\ =\left(\frac{\beta_{1}\eta }{a_{1}}-a_{2}\right)A_{1}-a_{4}A_{2}\\ =a_{2}\left(R_{1}-1\right)A_{1}-a_{4}A_{2}. \end{array}$$

By assumption *R*_1_ < 1, $\dot{V}\leq 0$. This implies that *V*(*X*) is a Lyapunov function with $\varrho =\frac{\eta }{a_{1}}$. Now, we apply the Lyapunov–LaSalle invariance principle. This indicates that the positive solutions tend to


$$\begin{array}{l} E=\left\{X\in {\mathbb{R}}_{+}^{5}|\dot{V}=0\right\}=\left\{\left(0,0,J_{2,}0,A_{3}\right)|J_{2},A_{3}\geq 0\right\}. \end{array}$$

Again, by applying the Bendixson–Dulac criteria to the limited *J*_2_*A*_3_-system of Equations 1–5, we observe that the non-trivial equilibrium is $\left(J_{2}^{*},A_{3}^{*}\right)$. Thus, the susceptible extinction equilibrium $\left(0,\;0,J_{2}^{*},0,A_{3}^{*}\right)$ is a global attractor of the system (Equations 1–5) in the domain ${\mathbb{R}}_{+}^{5}.$

The global stability of the susceptible extinction equilibrium implies the eventual elimination of all susceptible individuals, leaving only infected individuals. This potential outcome may arise if control measures are inadequately implemented or if the disease may propagate without restraint.

**Theorem 5**. *In the scenario of* *r* = ϵ = 1, *R*_1_ < 1*, and* *R*_0_ < 1*, we show that all solutions of the system* (Equations 1–5) *tend to* (0, 0, 0, 0, 0) *as time approaches infinity*.

Proof. Let us consider the positive definite function,


$$\begin{array}{l} V\left(X\right)=\rho_{1}J_{1}+A_{1}+\rho_{2}J_{2}+A_{2}+A_{3},\\ X=\left(J_{1},A_{1},J_{2},A_{2},A_{3}\right)\in {\mathbb{R}}_{+}^{5}. \end{array}$$

Setting $\rho_{1}=\frac{\eta }{a_{1}}$ and $\rho_{2}=\frac{\eta }{a_{3}}$, we have


$$\begin{array}{l} \dot{V}=\frac{\eta }{a_{1}}\left(\beta_{1}A_{1}-a_{1}J_{1}\right)+\left(\eta J_{1}-B\left(t\right)-a_{2}A_{1}\right)\\ +\frac{\eta }{a_{3}}\left(\beta_{2}A_{2}+\beta_{2}A_{3}-a_{3}J_{2}\right)+\left(B\left(t\right)-a_{4}A_{2}\right)+\left(\eta J_{2}+\theta A_{2}-a_{5}A_{3}\right)\\ =\left(\frac{\beta_{1}\eta }{a_{1}}-a_{2}\right)A_{1}+\left(\frac{\beta_{2}\eta }{a_{3}}+\theta -a_{5}-\alpha -\theta \right)A_{2}+\left(\frac{\beta_{2}\eta }{a_{3}}-a_{5}\right)A_{3}\\ =a_{2}\left(\frac{\beta_{1}\eta }{a_{1}a_{2}}-1\right)A_{1}+a_{5}\left(\frac{\beta_{2}\eta }{a_{3}a_{5}}-\frac{\alpha }{a_{5}}-1\right)A_{2}+a_{5}\left(\frac{\beta_{2}\eta }{a_{3}a_{5}}-1\right)A_{3}\\ \leq a_{2}\left(R_{1}-1\right)A_{1}+a_{5}\left(T_{2}-1\right)A_{2}+a_{5}\left(T_{2}-1\right)A_{3}. \end{array}$$

By our assumption *R*_1_ < 1, *R*_0_ < 1(⇒*T*_2_ < 1); therefore, $\dot{V}\left(X\right)\leq 0$. This shows that *V* is a Lyapunov function with the selected ρ_1_ and ρ_2_. Applying the Lyapunov–LaSalle invariance principle,


$$\begin{array}{l} E=\left\{X\in {\mathbb{R}}_{+}^{5}|\dot{V}=0\right\}=\left\{\left(0,0,0,0,0\right)\right\} \end{array}$$

where *E* is an invariant set. Thus, using the aforementioned principle, all positive solutions of the system (Equations 1–5) tend to *E* as *t* → 0.

It is also critical to see that all threshold values in our model are biologically meaningful. Table 1 summarizes all our major mathematical results for the system of Equations 1–5.

Theorem 1 proves the local stability of the disease-free equilibrium, i.e., if a ratio derived from the basic offspring number κ_1_ is >1, then we obtain a unique disease-free equilibrium.

Moreover, if the basic reproduction number *R*_0_ is <1 and κ_1_ is greater than the ratio $\frac{\eta \mu_{2}}{\left(\eta +\mu_{1}\right)\left(v_{1}+v_{2}+\mu_{2}\right)}$, which is clearly >1, i.e., if κ_1_ is greater than the inverse of the product of the probability of surviving in *J*_1_ and probability of dying in *A*_1_, then Theorem 3 shows that all infected classes will gradually cease to exist and only susceptible classes will approach a positive constant value.

If there is no recovery under any initial conditions and *R*_0_ ≥ 1, then Theorem 2 gives a unique susceptible extinction equilibrium. Moreover, if we consider all newborns from infected mothers to be infected, offspring number is <1, and $\kappa_{2}>\frac{\eta +\mu_{1}+\alpha }{\eta }$, then Theorem 4 shows that susceptible classes will eventually vanish over time and infected classes will approach constant values. Here, κ_2_ is derived from *T*_2_ = 1 and $\kappa_{2}>\frac{\eta +\mu_{1}+\alpha }{\eta }$. This means that κ_2_ is greater than the inverse of the probability of survival of *J*_2_.

By Theorem 5, if the basic offspring number is <1 and the sum of all ratios used in horizontal and vertical transmission of an infected individual is <1, then the total population will vanish over time. This means that if growth in susceptible and infected classes is not sufficient, then the whole population will become extinct.

However, as *r* = 1 and ϵ = 1 is not practically feasible, it is not possible to observe a susceptible extinction equilibrium. Meanwhile, if a sufficient growth rate is maintained, the solution to the system will not approach a trivial equilibrium; rather, it will approach a disease-free equilibrium provided that the basic reproduction number is below one. However, owing to several non-linearities in the model, we skip the tedious stability analysis of the endemic equilibrium; from the present analysis, it is straightforward to conclude that the basic reproduction number is the threshold for an outbreak. We implemented this model to explore the recent HIV scenarios in the USA and Pakistan.

## 4 Model implementation

For the experimental setup, we first describe the parameters and their values in Tables 2, 3. To integrate the system of differential Equations 1–5, some parameters were estimated with the help of data; the remaining ones were estimated using the maximum likelihood method.

**Table 2:** Parameters in our proposed model.

**Symbol**	**Description**	**Value (USA)**	**Value (Pakistan)**	**Unit**
β_1_	Per capita birth rate of susceptible adults	0.01192	0.029	*yr* ^−1^
*v*_2_	Transmission rate due to *A*_3_	1.55 × 10^−10^	0.0142	*yr* ^−1^
*r*	Probability of vertical transmission from *A*_2_	0.40	0.40	D.L
ϵ	Probability of vertical transmission from *A*_3_	0.1	0.1	D.L
α	Disease-induced death rate	1/19	1/13	*yr* ^−1^
μ_1_	Natural juvenile mortality rate	0.001541	0.0109	*yr* ^−1^
μ_2_	Natural adult mortality rate	1/65.57	1/51.7536	*yr* ^−1^
η	Maturation rate of juvenile population	1/13	1/15	*yr* ^−1^
*K*	Carrying capacity of population	645.42	407.39	Million
μ	Natural mortality rate	1/78.57	1/66.7536	*yr* ^−1^
*m*	Density-dependent death rate	$\frac{\beta_{1}-\mu }{K}$	$\frac{\beta_{1}-\mu }{K}$	*yr* ^−1^

All parameters were either estimated using data or taken from different references. Here, D.L denotes that the unit is dimensionless.

**Table 3:** Estimated parameters for the data of Pakistan and the USA.

**Symbol**	**Description**	**Value (CI) for the USA**	**Value (CI) for Pakistan**	**Unit**
β_2_	Per capita birth rate of infected adults	0.0011 (0.000923, 0.00130)	0.0274 (0.02637, 0.02851)	*yr* ^−1^
*v*_1_	Transmission rate due to *A*_2_	0.3045 (0.30104, 0.30794)	0.2559 (0.25446, 0.25738)	*yr* ^−1^
θ	Rate of screening and treatment	0.2495 (0.24604, 0.25286)	0.0182 (0.01760, 0.01872)	*yr* ^−1^

These parameters were estimated using the maximum likelihood method. Here, CI represents the confidence interval.

### 4.1 Parameterization for the USA

In the USA (47), the annual live infected births per thousand from 2014 to 2018 were recorded as 12.4, 12.2, 11.8, 11.6, and 11.6, respectively. Calculating the average value of β_1_ over this period yields


$$\begin{array}{l} \beta_{1}=\frac{11.92}{1000}=0.01192. \end{array}$$

According to the World Health Organization, if infected mothers are unaware of their infection, then the transmission rate of HIV infection from mother to newborn is between 15 and 45%. With awareness and treatment, this can be reduced to 5%. Therefore, we considered the values of *r* = 0.4 and ϵ = 0.1 for our model simulation.

To determine the transmission rate (*v*_2_) due to diagnosed infected adults, we used the estimated incidence rate for the USA in 2014, which was 14.3 per 100,000 (7). We also know that the diagnosed adult population of the USA was 926,209 in 2014, and the estimated total number of infected adults in 2010 was 1,085,100. The total population (adults) was 256,498,913; therefore, the susceptible adult population in 2014 was 255,413,813, and we calculated the value of transmission rate due to the diagnosed infected adults as follows:


$$\begin{array}{l} \frac{v_{2}A_{1}\left(2014\right)A_{3}\left(2014\right)}{A\left(2014\right)}=\text{incidence rate in }2014.\\ \text{This implies }v_{2}=\frac{14.3\times \left(256,498,913\right)}{\left(100,000\right)\left(255,413,813\right)\left(926,209\right)}\\ =1.55\times 10^{-10}. \end{array}$$

The average infection period of HIV in developed countries like the USA is reported to be between 8.6 and 19 years (27, 48). It was shown that the average infection period of HIV is 8.6 − 19 years. Given the excellent health facilities, we considered an average infection period of 19 years, resulting in a mean infection duration (MID) for the USA of $\alpha =\frac{1}{MID}=\;\frac{1}{19}$.

The UN Inter-agency Group for Child Mortality Estimation (49) provided the probabilities of dying in the first 5 years of life in the USA from 2014 to 2018. The average values (*JDR*_0 − 4_) for these years were calculated as:


$$\begin{array}{l} \text{JD}{\text{R}}_{0-4}=\frac{0.00688\;+\;0.0068\;+\;0.00673\;+\;0.00666\;+\;0.00658}{5}\\ =0.00673. \end{array}$$

Similarly, the estimated probabilities of dying between the ages of 5 and 15 for the same period were used to compute the average (*JDR*_5 − 14_), resulting in:


$$\begin{array}{l} \text{JD}{\text{R}}_{5-14}=\frac{0.0129\;+\;0.0131\;+\;0.0133\;+\;0.0135\;+\;0.0137}{5}\\ =0.0133. \end{array}$$

Consequently, the juvenile natural mortality rate (μ_1_) in the USA was determined as


$$\begin{array}{l} \mu_{1}=\frac{1}{13}\left(0.00673+0.0133\right)=0.001545 \end{array}$$

[see (27)]

The UN Inter-agency Group for Child Mortality Estimation (49) provided the probabilities of dying in the first 5 years of life in the USA from 2014 to 2018. The average values (*JDR*_0 − 4_) for these years were calculated as:


$$\begin{array}{l} \text{JD}{\text{R}}_{0-4}=\frac{0.00688\;+\;0.0068\;+\;0.00673\;+\;0.00666\;+\;0.00658}{5}\\ =0.00673. \end{array}$$

According to World Bank data, the average life expectancy (ALE) in the USA from 2014 to 2018 was 78.57 (50). The natural death rate μ is the inverse of ALE; therefore,


$$\begin{array}{l} \text{Natural mortality rate}=\mu =\frac{1}{\text{ALE}}=\frac{1}{78.57}\text{,} \end{array}$$

and


$$\begin{array}{l} \text{Natural mortality rate in adults }=\mu_{2}=\frac{1}{78.57-13}=\frac{1}{65.57} \end{array}$$

We consider the maximum age of the juvenile population as 13 years because the data were collected from the Centers for Disease Control and Prevention (CDC), which uses this cutoff (7, 51) therefore,


$$\begin{array}{l} \text{Maturation rate of the juvenile population}=\eta =\frac{1}{13} \end{array}$$

For the estimation of carrying capacity *C*, we considered the rate of change of the total population with respect to time as:


$$\begin{array}{l} g\left(N\right)=N^{\prime }=pN\left(C-N\right) \end{array}$$

Here, *p* is the kinetic parameter. Differentiating *g* with respect to *N*, we get


$$\begin{array}{l} \frac{dg}{dN}=p\left(C-2N\right) \end{array}$$

Because the function *g*(*N*) is bounded and increasing, by the first-order derivative criteria, we can obtain the maximum value of *N*_max_ = *C*/2 for this function. Thus,


$$\begin{array}{l} g_{\text{max}}=p\frac{C}{2}\left(C-\frac{C}{2}\right)=p\frac{C^{2}}{4} \end{array}$$

and with the help of Table 4, we get *g*_max_ = 2, 334, 155, where

**Table 4:** Total populations of the USA and Pakistan from 2014 to 2018.

**Year**	**2014**	**2015**	**2016**	**2017**	**2018**
Total population of USA	318,301,008	320,635,163	322,941,311	324,985,539	326,687,501
Total population of Pakistan	195,306,825	199,426,964	203,627,284	207,896,686	212,215,030

These time-series data for the US and Pakistan populations were taken from the World Bank (52).

*g*_max_ = max|*difference of two consecutive years* |.

The average total population of the USA (2014–2018) was *N* = 322, 710, 104.


$$\begin{array}{c} g_{\max}=pN\left(C-N\right)\\ \text{Implies }2,334,155=p\left(322,710,104\right)\left(C-322,710,104\right)\\ p=\frac{4,318,344}{\left(322,710,104\right)\left(C\;-\;322,710,104\right)}.\\ \text{Moreover, }g_{\max}=\frac{pC^{2}}{4}.\\ \text{Implies }C^{2}=\frac{4g_{max}}{p}=\frac{4\left(4,318,344\right)\left(322,710,104\left(C\;-\;322,710,104\right)\right)}{4,318,344}\\ =4\left(322,710,104\left(C-322,710,104\right)\right).\\ \Rightarrow \;C^{2}-4\left(322,710,104\right)C+4{\left(322,710,104\right)}^{2}=0 \end{array}$$

This implies that the average value of *C* is 645.42 million.

Initial values for the simulation were given as follows:

*J*_1_(0) = *Total juvenile population* − *J*_2_(0) = 61, 802, 095−(2, 477+2, 477 × 0.14) is the approximate initial susceptible juvenile population in 2014 in the USA (52, 53).

*A*_1_(0) = *A*(0) − *Total adult infected population* = 256, 498, 913 − 10, 851, 00 is the approximate initial susceptible adult population in 2014 in the USA (7, 52, 53).

*J*_2_(0) = 2, 477+2, 477 × 0.14 is the initial HIV-infected juvenile population in 2014 in the USA (51).

*A*_2_(0) = 1, 085, 100 − 926, 209 is the initial HIV-infected adult population not in treatment in 2014 in the USA (7, 51).

*A*_3_(0) = 926, 209 is the initial HIV-infected adult population in treatment in 2014 in the USA (51).

### 4.2 Parameterization for Pakistan

In the study of the World Bank (47), the numbers of live-infected births each year (per thousand) from 2014 to 2018 in Pakistan were 29.318, 29.124, 28.888, 28.599, and 28.25, respectively. We consider the same values of *r* and ϵ for Pakistan as we did for the USA.

From UNAIDS (25), we estimated that the average number of newly infected adults was 18,000 per year from 2014 to 2018. The average percentage of people in treatment was 10% (6) and the average total infected during this time interval per year in Pakistan was 126,800 (54). We assume the same percentage of newly aware infected adults in treatment. Therefore, we use the formula given by López et al. (27) as follows:


$$\begin{array}{c} v_{2}=\frac{\text{Average total new infected adults }\left(\text{per year}\right)\times 10\%}{\text{Average total infected population }}\\ =\frac{\left(18,000\right)\left(0.1\right)}{126,800}\approx 0.0142 \end{array}$$

Once an individual is infected with HIV, the average infection period of HIV is 8.6–19 years (27, 48). Because Pakistan is a developing country, we consider the average life expectancy after infection of 13 years as the MID for our model. Thus,


$$\begin{array}{c} \alpha =\frac{1}{\text{MID}}=\frac{1}{13} \end{array}$$

According to the UN Inter-agency Group for Child Mortality Estimation, the probabilities of dying in the first 5 years of life from 2014 to 2018 were 0.0783, 0.07605, 0.07381, 0.07158, and 0.0694, respectively.

Therefore, the average value (*JDR*_0 − 4_) is:


$$\begin{array}{c} \text{JD}{\text{R}}_{0-4}=\frac{0.0783\;+\;0.07605\;+\;0.07381\;+\;0.07158\;+\;0.0694}{5}\\ =0.073828. \end{array}$$

In addition, the probabilities of dying between the ages of 5 and 15 from 2014 to 2018 were 0.0939, 0.0918, 0.0897, 0.0875, and 0.0856, respectively (55).

Therefore, the average value (*JDR*_5 − 14_) is:


$$\begin{array}{c} \text{JD}{\text{R}}_{5-14}=\frac{0.0939\;+\;0.0918\;+\;0.0897\;+\;0.0875\;+\;0.0856}{5}\\ =0.0897. \end{array}$$

Thus, the juvenile natural mortality rate is:


$$\begin{array}{c} \mu_{1}=\frac{1}{15}\left(0.073828+0.0897\right)=0.0109. \end{array}$$

The average life expectancy in Pakistan from 2011 to 2018 was 66.7536 (50). The natural death rate μ is the inverse of this; therefore,


$$\begin{array}{c} \text{Natural mortality rate}=\mu =\frac{1}{66.7536}\text{,} \end{array}$$

and


$$\begin{array}{c} \text{Natural mortality rate of adults }=\mu_{2}=\frac{1}{66.7536-15}=\frac{1}{51.7536}\text{.} \end{array}$$

Considering the maximum age of the juvenile population as 15 years (27, 48),


$$\begin{array}{c} \text{Maturation rate of the juvenile population}=\eta =\frac{1}{15}. \end{array}$$

For carrying capacity *C*, we used the same estimation approach as for the US data.

As Table 4 shows, *g*_max_ = 43, 183, 44. From the same table, the average total population of Pakistan (2014-2018) was *N* = 203, 694, 557.8. Therefore,


$$\begin{array}{c} C^{2}=\frac{4\left(4,318,344\right)\left(\left(203,694,557.8\right)\left(C-203,694,557.8\right)\right)}{4,318,344}\\ =4\left(\left(203,694,557.8\right)\left(C-203,694,557.8\right)\right)\\ \Rightarrow C^{2}-4\left(203,694,557.8\right)C+4{\left(203,694,557.8\right)}^{2}=0. \end{array}$$

This implies that the average value of *C* is 407.39 million.

Initial values for the simulation were given as follows:

*J*_1_(0) = *Total juvenile population* − *J*_2_(0) = 70, 797, 567 − 3, 500 is the approximate initial susceptible juvenile population in 2014 in Pakistan (25, 53).

*A*_1_(0) = *A*(0) − *A*_2_(0) − *A*_3_(0) = 124, 509, 258 − 90, 500 is the approximate initial susceptible adult population in 2014 in Pakistan (25, 52–54).

*J*_2_(0) = 3, 500 is the initial HIV-infected juvenile population in 2014 in Pakistan (25).

*A*_2_(0) = 90, 500 − 6, 292 is the initial HIV-infected adult population not in treatment in 2014 in Pakistan (54).

*A*_3_(0) = 6, 292 is the initial HIV-infected adult population in treatment in 2014 in Pakistan (54).

### 4.3 Maximum likelihood fitting

In this subsection, we explain the maximum likelihood method and estimation of the remaining parameters. Because β_2_ the birth rate and *v*_1_ the transmission rate due to infected adults, *A*_2_ and the status of infection are not always known, estimating them directly from data of newborns and the newly infected is not convenient. Instead, we estimate the parameters β_2_, *v*_1_, and θ using the maximum likelihood method. We have time-series data of juvenile infected, HIV-positive adults in treatment and infected population not in treatment (total infected – infected in treatment; see Tables 5, 6).

**Table 5:** HIV-diagnosed adult and juvenile populations and total adult infected populations in the USA from 2014 to 2018.

**Year**	**2014**	**2015**	**2016**	**2017**	**2018**
HIV-positive in treatment	926,209	951,346	976,097	1,000,062	1,023,832
Juvenile diagnosed HIV+	2,477	2,344	2,226	2,072	1,912
Total adult HIV+	1,085,100	1,108,400	1,131,100	1,152,500	1,173,900

Because the USA is a developed country, we assume that all diagnosed infected in the USA are in treatment. This time-series data for HIV-diagnosed adult population are from the CDC (51). Because 14% of HIV-infected population do not know their status of infection (7), we add this population to the diagnosed juvenile population and assume that this is the total infected juvenile population in the USA. The time-series data for HIV-diagnosed juvenile population are from the CDC (51). The time-series data for total estimated adult HIV-infected population are from the CDC (7).

**Table 6:** HIV-positive (aware and in treatment) adult, juvenile infected, and total infected population in Pakistan from 2014 to 2018.

**Year**	**2014**	**2015**	**2016**	**2017**	**2018**
HIV-positive in treatment	6,292	6,500	8,900	12,046	15,821
Juvenile HIV+	3,500	4,000	4,500	5,000	5,500
Total HIV+	94,000	100,000	130,000	150,000	160,000

This time-series data for HIV-diagnosed adult population are from UNAIDS and the AIDS Data Hub (54). The time-series data for HIV juvenile population are from UNAIDS estimation (2), and the time-series data for estimated total HIV-infected population are from UNAIDS and the AIDS Data Hub (54).

Let $X=\left(J_{1},A_{1},J_{2},A_{2},A_{3}\right)\in {\mathbb{R}}_{+}^{5}$ be the vector of state variables and 𝔽 be the right side of the system (Equations 1–5). ℙ = (β_2_, *v*, θ) is the vector of parameters to be estimated. 𝕐(*t*, ℙ) = (*A*_3_(*t*, ℙ), *J*_2_(*t*, ℙ), *A*_2_(*t*, ℙ)) is the vector of observables. 𝕐^0^(*t*, ℙ) represent the observed data at *t* = 2014, ⋯ , 2018. We assume that all 𝕐^0^(*t*, ℙ) are independent and Poisson distributed with mean 𝕐(*t*, ℙ). Thus, the Poisson maximum likelihood function will be:


$$\begin{array}{c} \text{L}\left({𝕐}^{0}\left(t,\mathbb{P}\right)|𝕐\left(t,\mathbb{P}\right)\right)=\overset{3}{\underset{j=1}{\prod }}\overset{5}{\underset{i=1}{\prod }}\frac{y_{j}{\left(t_{i}\right)}^{y_{ji}^{0}}\cdot e^{-y_{j}\left(t_{i}\right)}}{y_{ji}^{0}!}. \end{array}$$

Therefore, the negative log-likelihood function (NLF) reduces to


$$\begin{array}{c} \text{NLF}=-\ln\;\left(\overset{3}{\underset{j=1}{\prod }}\overset{5}{\underset{i=1}{\prod }}\frac{y_{j}{\left(t_{i}\right)}^{y_{ji}^{0}}\cdot e^{-y_{j}\left(t_{i}\right)}}{y_{ji}^{0}!}\right)\\ =-\overset{3}{\underset{j=1}{\sum }}\overset{5}{\underset{i=1}{\sum }}y_{ji}^{0}\ln\;\left(y_{j}\left(t_{i}\right)\right)+\overset{3}{\underset{j=1}{\sum }}\overset{5}{\underset{i=1}{\sum }}y_{j}\left(t_{i}\right)+\overset{3}{\underset{j=1}{\sum }}\overset{5}{\underset{i=1}{\sum }}\ln\;\left(y_{ji}^{0}!\right). \end{array}$$

Because the last term in the aforementioned equation is constant, it will remain unchanged as the parametric values are varied. Therefore, we can minimize only the first two terms of the equation. Hence, the fitting problem can be expressed as


$$\begin{array}{c} \text{min}\left(\text{NLF}\right)=\text{min}\left(-\overset{3}{\underset{j=1}{\sum }}\overset{5}{\underset{i=1}{\sum }}y_{ji}^{0}\ln\;\left(y_{j}\left(t_{i}\right)\right)+\overset{3}{\underset{j=1}{\sum }}\overset{5}{\underset{i=1}{\sum }}y_{j}\left(t_{i}\right)\right) \end{array}$$

subject to


(7) $$\begin{array}{c} \frac{d}{dt}X\left(t,\mathbb{P}\right)=F\left(X,\mathbb{P},t\right)\\ 𝕐\left(t,\mathbb{P}\right)=\left(A_{3}\left(t,\mathbb{P}\right),J_{2}\left(t,\mathbb{P}\right),A_{2}\left(t,\mathbb{P}\right)\right)\\ X\left(0\right)=\left(J_{1}\left(2014\right),A_{1}\left(2014\right),J_{2}\left(2014\right),A_{2}\left(2014\right),A_{3}\left(2014\right)\right)\\ X\left(t,\mathbb{P}\right),\mathbb{P}\geq 0. \end{array}$$

The aforementioned minimization problem will provide the desired fitting with practically feasible values of parameters if ℙ is identifiable, which can be confirmed in two steps. First, we examine the structural identifiability, and then we confirm the practical identifiability.

Structural identifiability refers to the existence of a unique solution to *X*(*t*, ℙ) for each ℙ under an initial condition. If any component of ℙ is implicitly related, different values of ℙ may yield the same solution *X*(*t*, ℙ) for a given initial condition, which might hinder the unique estimation of parameters ℙ for the data.

We use the Fisher information matrix (FIM) to confirm the structural identifiability. We have a set of observations at five distinct points, a system represented by a five-dimensional state vector, and a three-dimensional vector of parameters. Thus, the sensitivity matrix *S* consists of five time-dependent 5 × 3 blocks 𝔸(*t*_*n*_):


$$\begin{array}{c} S=\left[\begin{array}{c} 𝔸\left(t_{1}\right)\\ 𝔸\left(t_{2}\right)\\ \vdots \\ 𝔸\left(t_{5}\right) \end{array}\right] \end{array}$$

where


$$\begin{array}{c} {𝔸}_{jk}\left(t_{n}\right)=\frac{\partial x_{j}\left(t_{n},\mathbb{P}\right)}{\partial P_{k}},\;n=1,\cdots \;,5,\;k=1,2,3\text{ and }j=1,\cdots \;,5. \end{array}$$

The 3 × 3 FIM is *M* = *S*^*T*^*S*. The rank of the matrix *M* counts the number of identifiable parameter conditions in ℙ, and the parameters in ℙ are structurally identifiable if and only if *M* has rank 3.

According to the aforementioned definition, the FIM for our problem has three columns, which correspond to the parameters to be estimated. Let us denote the parameter estimates by $\hat{\beta_{2}},\hat{v_{1}}$, and $\hat{\theta }$. To approximate the FIM numerically, we perturb $\hat{\beta_{2}}$ to the new values $\hat{\beta_{2}^{+}}=\left(1+0.001\right)\hat{\beta_{2}}\;and\;\hat{\beta_{2}^{-}}=\left(1-0.001\right)\hat{\beta_{2}}$, for which we integrate the model for each observation time. Then, we numerically approximate the derivatives, $A_{j1}\left(t_{n}\right)=\frac{\partial x_{j}\left(t_{n},\hat{\beta_{2}},\hat{v_{1}},\hat{\theta }\right)}{\partial \hat{\beta_{2}}},n=1,\cdots \;,5,j=1,\cdots \;,5$. This gives the first column. Meanwhile, the other two variables $\hat{v_{1}}$ and $\hat{\theta_{2}}$ remain fixed. We repeat the same procedure for $\hat{v_{1}}$ and $\hat{\theta_{2}}$ to obtain the second and third columns, respectively. Then, we check the rank of matrix *M*, which is 3. This ensures the structural identifiability of the parameters.

Practical identifiability or “estimableness” refers to the sufficiency of available observations, as too few observations might be insufficient for fitting. To investigate the practical identifiability, we compute the profile likelihood of the parameters β_2_, *v*_1_, and θ_2_. Profile likelihood reveals the dependency of the NLF on individual parameters, which helps us to find finite confidence intervals for each parameter; otherwise, practical non-identifiability is proved. The related profile likelihoods can be defined as


$$\begin{array}{c} PL_{\beta_{2}}\left(\beta_{2}\right)=\underset{v_{1},\theta }{\min}\left\{\text{NLF}\left(\beta_{2},v_{1},\theta \right)\right\}, \end{array}$$

$PL_{v_{1}}\left(v_{1}\right)=\underset{\beta_{2},\theta }{\min}\left\{\text{NLF}\left(\beta_{2},v_{1},\theta \right)\right\}$, and $PL_{\theta }\left(\theta \right)=\underset{\beta_{2},v_{1}}{\min}\left\{\text{NLF}\left(\beta_{2},v_{1},\theta \right)\right\}$.

where $\beta_{2}\in \left[\hat{\beta_{2}}\left(1-0.001\right),\hat{\beta_{2}}\left(1+0.001\right)\right]$, $v_{1}\in \left[\hat{v_{1}}\left(1-0.001\right),\hat{v_{1}}\left(1+0.001\right)\right]$, and


$$\begin{array}{c} \theta \in \left[\hat{\theta }\left(1-0.001\right),\hat{\theta }\left(1+0.001\right)\right] \end{array}$$

To determine the confidence interval, we have $2\left(\text{NLF}\left(\mathbb{P}\right)-\text{NLF}\left(\hat{\mathbb{P}}\right)\right)\sim \chi_{3}^{2}$. Therefore, the NLF threshold for a 95% confidence interval is $\text{NLF}\left(\hat{\mathbb{P}}\right)+7.815/2$.

**Figure 2:**
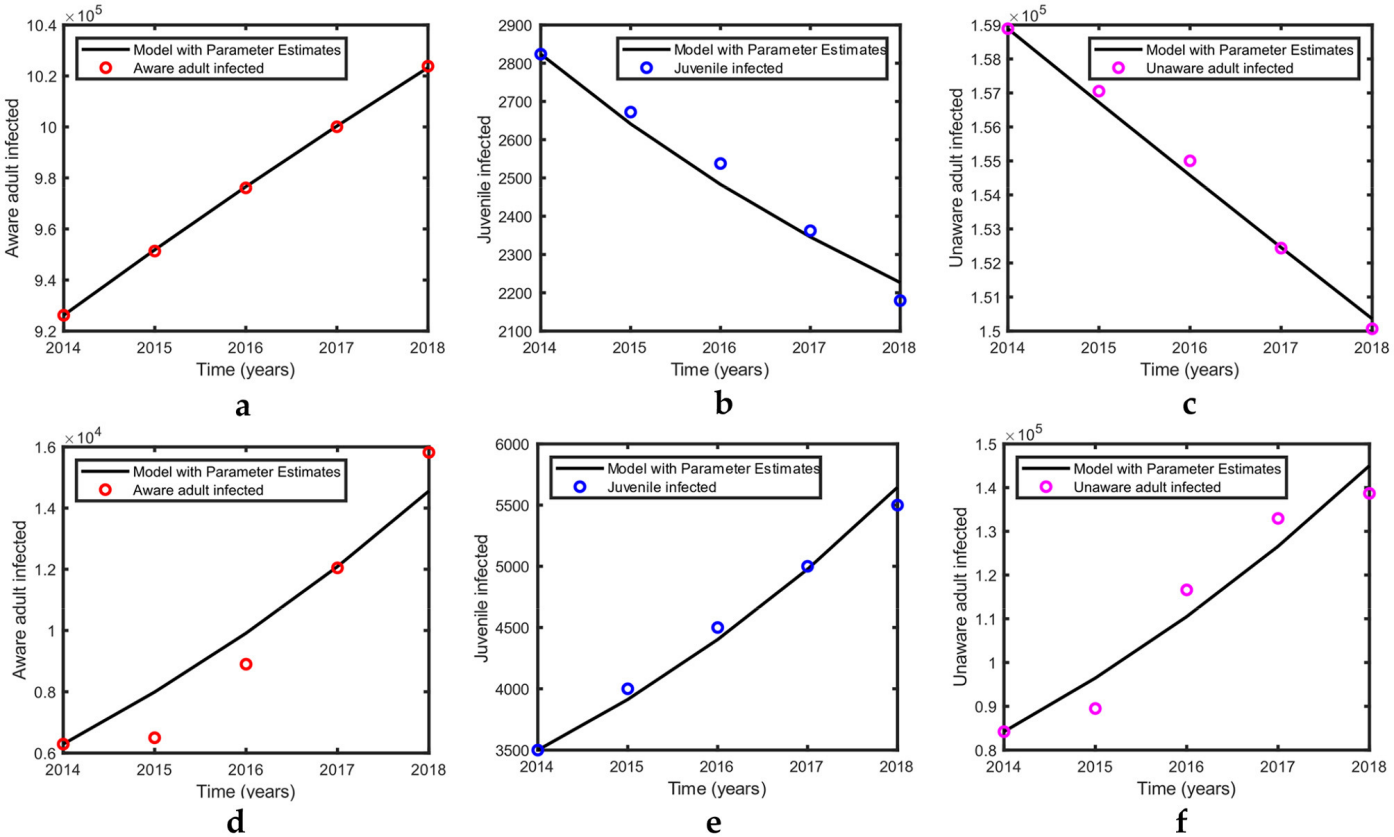
Simulation results for US and Pakistan data. Because the USA is a developed country, we assume that all diagnosed infected in the USA are in treatment. The parametric values for the USA in Tables 2, 3 were used in this simulation, whereas the initial values are defined in Section 4.1. **(a–c)** show the curves fitted with US data. Parametric values for Pakistan in Tables 2, 3 were used in this simulation, whereas the initial values are defined in Section 4.2. **(d–f)** show the curves fitted with Pakistan data.

The maximum likelihood fitting of the time-series data for the USA and Pakistan is shown in Figure 2; the corresponding estimates of the parameters are shown in Table 3 along with the 95% confidence intervals. The fittings for both the USA and Pakistan appear relatively good at a glance. Moreover, the estimated parameter values are biologically meaningful. Because the birth rate of an infected population is less than that of a susceptible adult, we observe that $\beta_{1}>\hat{\beta_{2}}$ in the estimation. In addition, $\hat{v_{1}}>v_{2}$ due to awareness and treatment. When a person is aware of their infection, the transmission rate may be reduced because of treatment and precautions.

**Figure 3:**
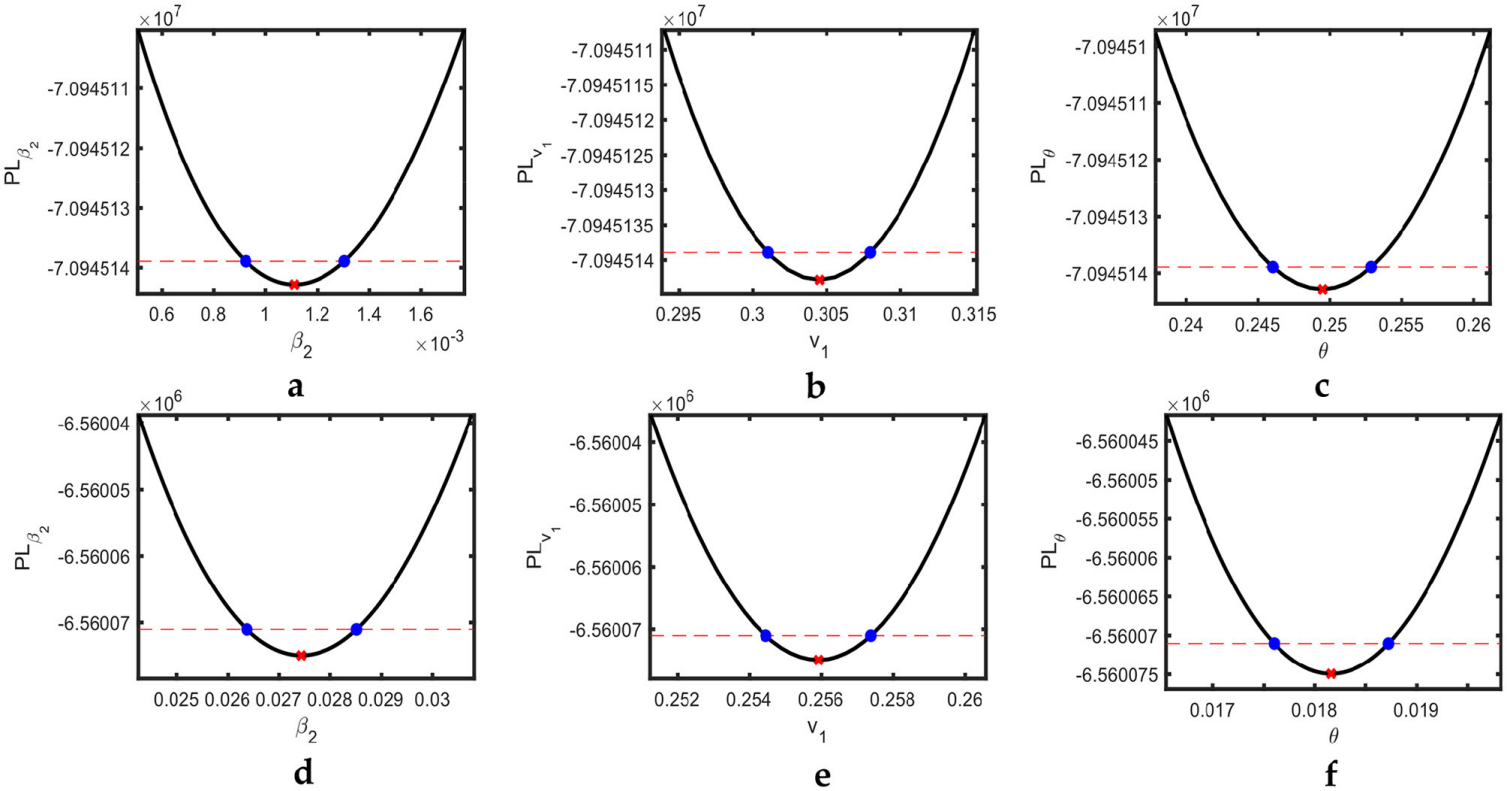
Estimated parameters versus profile likelihood function. **(a–c)** Show graphs of profile likelihood (cost) functions and estimated parameters β_2_, *v*_1_, and θ for the USA, respectively, and **(d–f)** show the same for Pakistan. Red crosses indicate the estimated values of the parameters, whereas blue dots show the confidence intervals.

Profile likelihoods for the USA and Pakistan are shown in Figure 3, revealing the minimization of the NLF at the estimated values of the parameters while also confirming the practical identifiability. The solid black lines indicate the cost as a function of β_2_, *v*_1_, and θ. For each cost function, we change the value of one parameter using the minimization algorithm; the others vary on the parallel axis over the interval shown. This pattern of the cost function verifies the identifiability criteria of our estimated parameters. The red cross on each curve shows the best-fitted value for the parameters β_2_, *v*_1_, and θ.

### 4.4 Sensitivity analysis

Sensitivity indices were used to compute how minor variations in the parameters of interest cause variability in the quantities of interest (56). With the help of the normalized sensitivity index (NSI) of *R*_0_ regarding the parameters, we can determine which parameters have a greater impact on disease transmissibility. The NSI of the basic reproduction number *R*_0_ to the parameter ϕ is defined as


$$\begin{array}{c} \frac{\partial R_{0}}{\partial \phi }\times \frac{\phi }{R_{0}}, \end{array}$$

where *R*_0_ is the quantity of interest and ϕ is our parameter of interest. Table 7 lists the NSIs of *R*_0_ with respect to the parameters for our current estimate.

**Table 7:** NSIs of basic reproduction number.

**Parameters**	**NSI (USA)**	**NSI (Pakistan)**
β_1_	−0.0333	−0.1907
*r*	1.1485 × 10^−11^	0.0046
ϵ	5.9286 × 10^−11^	0.0010
μ_1_	6.8130 × 10^−4^	0.0250
η	6.8328 × 10^−4^	−0.0321
β_2_	7.0771 × 10^−11^	0.0057
*v*_1_	1	0.9483
*v*_2_	1.0511 × 10^−8^	0.0461
μ_2_	−0.0067	−0.0443
θ	−0.7938	−0.1110
α	−0.1675	−0.6469
*m*	0	0

This table lists the sensitivity indices of the basic reproduction number *R*_0_ to the parameters used in the mathematical model (Equations 1–5) at the parametric values in Tables 2, 3. Here, NSI stands for local sensitivity index.

According to our analysis, the parameters with comparatively higher sensitivity are β_1_, *v*_1_, θ, and α. Negative signs of local sensitivity indices indicate that if we increase the value of these parameters of interest, then our quantity of interest will decrease, and vice versa. In short, *R*_0_ reduces with an increase in awareness and treatment rate θ.

## 5 Results

According to our estimation, the values of *R*_0_ for the USA and Pakistan are 0.9688 and 2.2599, respectively, which reveals that according to our analysis, HIV is approaching eradication in the USA. This is in agreement with the recent trend of decreasing incidence in the USA (Figure 2). Meanwhile, in Pakistan, the recent trend of increasing incidence (Figure 2) is reflected in the high estimate of the reproduction number.

**Figure 4:**
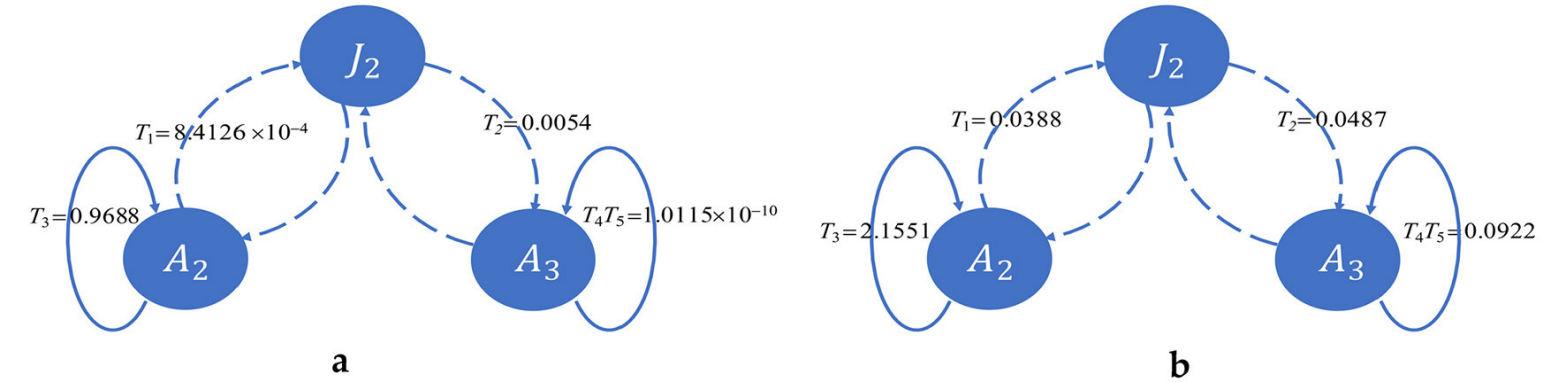
Contributions of horizontal and vertical transmissions to *R*_0_. Solid lines indicate horizontal transmission, and dotted lines indicate vertical transmission. Horizontal transmission from *A*_2_ plays a major role in both cases. **(a)** Shows transmission in the USA, whereas **(b)** shows transmission in Pakistan.

We have two infectious classes, *A*_2_ and *A*_3_, which each transmit the disease vertically and horizontally, constituting four transmission pathways. We split the contributions of these pathways to *R*_0_ in Figure 4.

This shows that horizontal transmission from *A*_2_ plays a vital role in both countries. In the case of the USA, *T*_3_ ≈ *R*_0_, which means the incidences in the USA are almost entirely due to adults being unaware of infection, whereas transmission due to other pathways is negligible. However, in the case of Pakistan, although horizontal transmission from *A*_2_ plays a major role, transmissions through the other three pathways also play a noticeable role. Transmissions through these three pathways are several times greater than in the USA. Moreover, the value of *v*_2_ is higher in Pakistan than in the USA. That is, individuals who know their status of infection and maintain treatment in the USA are more vigilant than those in the same class in Pakistan. On the contrary, *T*_3_ is higher and *v*_1_ is lower in Pakistan than in the USA, which indicates that individuals who are unaware of their infection status in Pakistan transmit less than those in the USA.

**Figure 5:**
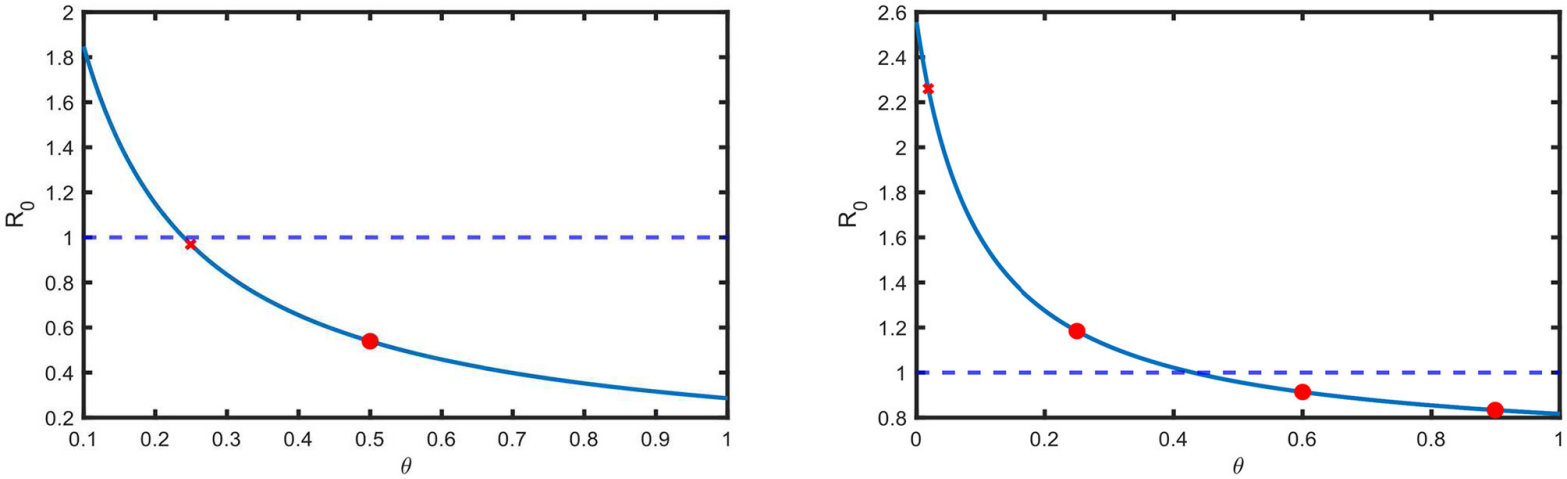
Basic reproduction number versus screening rate in the USA and Pakistan. The red crosses indicate the estimated value of the screening and treatment rate θ. Other red circles indicate that if we increase θ, then *R*_0_ decreases.

*R*_0_ has a negative sensitivity to the parameter θ (Table 7), which is related to awareness and treatment. This is also portrayed in Figure 5 for the estimated values of the parameters for both the USA and Pakistan. Here, the red crosses show the estimated values of θ, and the blue lines show the dependency of *R*_0_ on θ. According to Figure 5, *R*_0_ is below one if θ is above a threshold, which is 0.2396 and 0.4298 in the USA and Pakistan, respectively.

In the case of the USA, we observe that the estimated value of θ is 0.2495, which means that the disease is diagnosed within 4 years ($\frac{1}{0.2495}\approx 4$) of infection. This is above the threshold and has recently resulted in a declining prevalence in the USA. At present, *R*_0_ is very close to 1 in the USA, which implies that the disease will be eradicated, but it will take a long time. In the case of Pakistan, the estimated value of θ is below the threshold (Figure 5), and as a result, the prevalence has an increasing trend (Figure 2).

If the unaware infected population learn their status of infection and they are in treatment within ~2 years ($\frac{1}{\theta }$ $\leq \frac{1}{0.4302}$) after infection, then the disease will gradually be eradicated.

**Figure 6:**
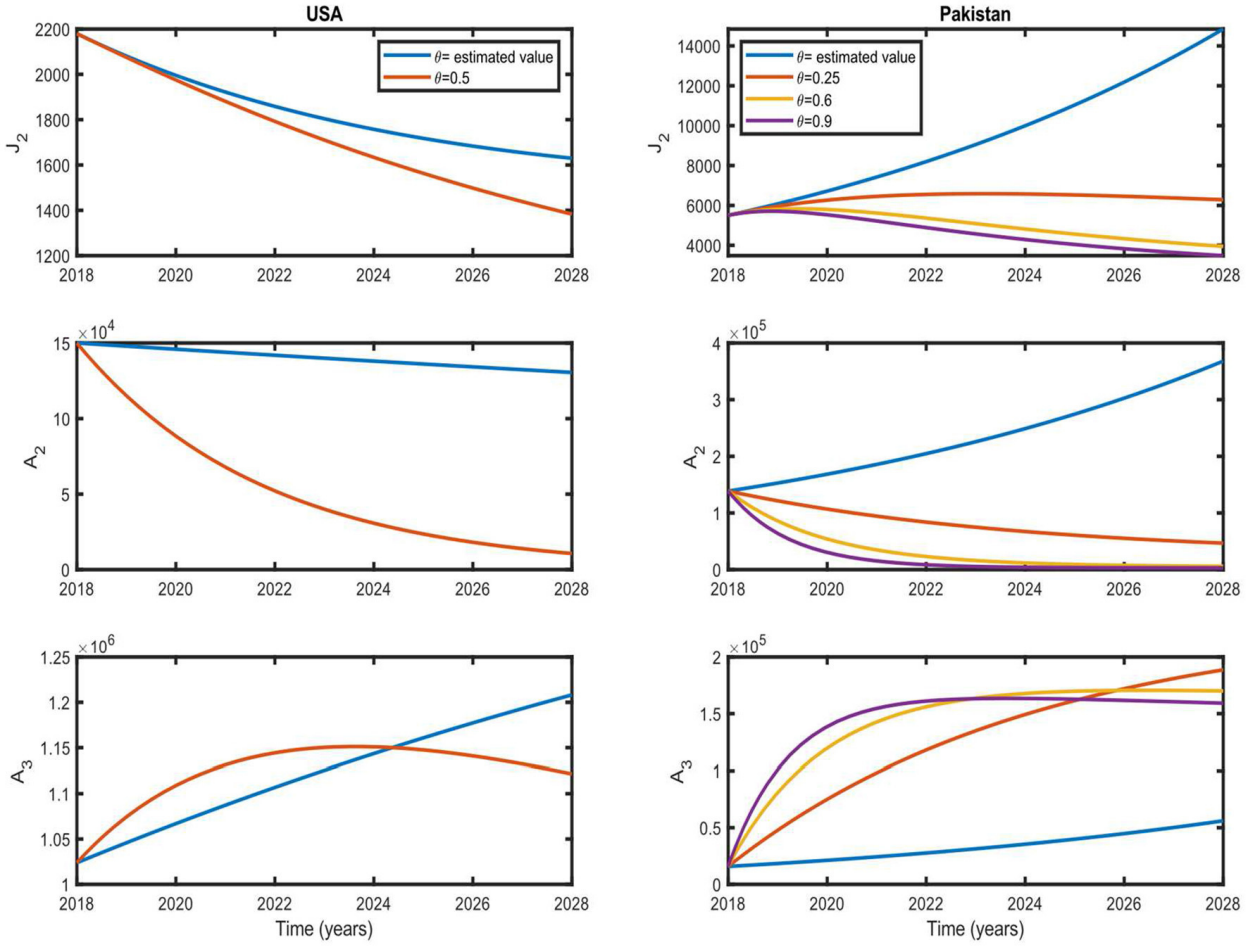
Simulation results with different values of treatment rate. These results show the situation of the infected population for the next 10 years with different values of θ. Initial values are US and Pakistan data from 2018 (see Tables 4–6).

To clarify the role of θ in disease prevalence, we simulated our model for different values of θ while keeping other parameters fixed within a time interval of 10 years, and the results are shown in Figure 6. For the US case, we show the simulation results for the estimated value of θ and θ = 0.5 (these values and corresponding *R*_0_ are shown in Figure 5 by a cross and dot, respectively). For the estimated value of θ, the number of individuals in the *J*_2_ and *A*_2_ classes decrease monotonically, whereas it increases for *A*_3_. For θ = 0.5, the number of individuals in all infected classes decreases monotonically except for *A*_3_.

*A*_3_ shows an increasing trend up to ~5 years due to increasing awareness and seeking treatment, and then it starts to decrease. In the case of Pakistan, we show the simulation results for the estimated value of θ, where θ = 0.25, 0.6, 0.9 (these values and corresponding *R*_0_ are shown in Figure 5 by a cross and dots, respectively). For the estimated value of θ, the number of individuals in all infected classes increases monotonically. If θ = 0.6, a decreasing trend in all the infected classes could be achieved after ~9 years. A decreasing trend could be achieved in ~5 years if θ is as high as 0.9.

If we increase the coverage of treatment, the pace of disease eradication will also increase. Therefore, screening and treatment can help control the spread of the disease in Pakistan and accelerate eradication in the USA.

## 6 Discussion

The HIV epidemic presents a distinct contrast between the USA and Pakistan. While the USA has made significant strides in controlling the spread of HIV, with continuous efforts in treatment and screening. Pakistan has seen a concerning in HIV in recent years. However, because of consistent improvement in healthcare infrastructure and availability of antiretroviral therapy (ART), infected people can lead an almost normal life with careful medication and a punctilious lifestyle (39). This shift in treatment efficacy addresses the concern raised decades ago that extending the lifespan of HIV patients could exacerbate the epidemic by increasing the basic reproduction number (*R*_0_) (27). To understand the role of treatment with improved medication in today's world, we investigated the recent HIV trends in the USA and Pakistan using a mathematical epidemic model.

HIV treatment brings the infected person closer to a normal life from several perspectives. For instance, it (1) reduces the fatality of the infection and increases lifespan, (2) reduces the probability of transmission to partners, and (3) reduces the probability of vertical transmission to children. To consider all these aspects, we assumed a two-age group model. The mathematical analysis of our model reveals that the basic reproduction number is the key threshold for an outbreak. The model is defined by several parameters and estimating all of them by fitting with time-series data of infected individuals leads to identifiability issues. Hence, we determined the values of most parameters from available literature and estimated the key parameters, birth rate of infected adults, transmission rate for infected not in treatment, and rate of screening and treatment, by fitting with time-series data.

The central finding of our research emphasizes the significance of the basic reproduction number (*R*_0_) as a key determinant in assessing the severity of an outbreak. The model parameters, which include birth rates, transmission rates, and treatment initiation rates, are crucial in predicting disease progression. Identifying all parameters from data is fraught with challenges, primarily stemming from issues of identifiability. To address this issue, we employed a combination of literature-based parameters and estimation methods to determine crucial variables, such as the infection rate of adults, transmission rates among untreated individuals, and the rates of screening and treatment initiation. Based on our findings, the USA should focus on expanding treatment coverage, while Pakistan should emphasize the promotion of preventive strategies and raising awareness.

First, we ensured the *structural identifiability* of these key parameters with respect to the model set by checking the rank of the FIM. Next, using the profile likelihood of the estimates, we confirmed the *practical identifiability* of the parameters for the time-series data of the infected. According to our estimates, the birth rate of infected individuals is lower than that of susceptible individuals. In addition, our estimation shows that treatment reduces the probability of both vertical and horizontal transmission, which indicates the role of treatment in HIV infection in both countries.

Furthermore, the reproduction number (*R*_0_) shows high sensitivity to transmission rate from infected individuals not in treatment and the rate of undergoing treatment following screening. This highlights the critical importance of *screening and awareness programs*. As infected individuals not in treatment are mostly not aware of their infection, screening is a potential measure to impede the incidence. This is also evident from the transmission pathway-wise splitting of the reproduction number; transmission from infected individuals not in treatment is the dominant factor in both the USA and Pakistan. However, infected individuals in treatment seem more mindful in the USA than in Pakistan. Moreover, the screening rate is better in the USA than in Pakistan.

Given these findings, we emphasize that increased screening and awareness campaigns could play an important role in controlling the HIV epidemic in Pakistan. Although the USA has made substantial progress, improving the rate of screening further could still yield significant reductions in HIV incidence. Our model suggests that by identifying infected individuals and putting them in treatment within ~2 years in the USA and 1.1 years in Pakistan, it is possible to achieve a declining trend within ~5 years in all infected classes.

## 7 Conclusion

In this article, we proposed a two-age group model to differentiate between the transmissibility of a juvenile and two adult infected classes. We fitted it with time-series data of infected individuals from the USA and Pakistan. According to our estimation, the basic reproduction numbers for the USA and Pakistan are 0.9688 and 2.2599, respectively. In addition, the estimated values of the parameters show that the birth rate of infected individuals is lower than that of susceptible individuals, and the transmission rate due to the infected not undergoing treatment is higher than that of infected individuals in treatment, which demonstrates the consistency of our approach. The basic reproduction number is most sensitive to the transmission rate from infected individuals not in treatment and the rate at which these individuals are screened and given treatment. Furthermore, by splitting the roles of the different transmission pathways, it became apparent that the infected group not in treatment contributes the most to transmission in both countries. Therefore, screening and treatment will reduce the most transmissible group and work in a bi-directional manner to reduce the incidence rate, which we also confirmed through appropriate simulations of our model.

A comparison of the parameter values for the USA and Pakistan showed that the infected individuals in treatment in the USA are more inclined to follow the guidelines or take better care than those in Pakistan. On the contrary, infected individuals not in treatment transmit the disease in the USA more than those in Pakistan. Although the USA already has a healthy rate of screening and treatment, improvement in the rate is necessary for prompt eradication of the disease. However, besides screening and treatment, mass education and awareness are crucial in Pakistan.

Our study inevitably had some limitations because of certain assumptions. Different stages of HIV infection have different degrees of transmissibility. Therefore, modeling stage-dependent infectiousness may help identify the most effective target group for screening. Furthermore, considering a two-sex model would facilitate understanding and reduce vertical transmission, as the probability of mother-to-child transmission is much higher than that of father-to-child transmission. Future research considering the aforementioned factors could be useful for further enhancement of control strategies.

## Data Availability

The original contributions presented in the study are included in the article/supplementary material, further inquiries can be directed to the corresponding author.
